# Hybrid systems using residual modeling for sea surface temperature forecasting

**DOI:** 10.1038/s41598-021-04238-z

**Published:** 2022-01-11

**Authors:** Paulo S. G. de Mattos Neto, George D. C. Cavalcanti, Domingos S. de O. Santos Júnior, Eraylson G. Silva

**Affiliations:** Centro de Informática, Universidade Federal de Pernambuco, Recife, Pernambuco Brazil

**Keywords:** Atmospheric science, Climate change, Climate and Earth system modelling, Projection and prediction, Computer science

## Abstract

The sea surface temperature (SST) is an environmental indicator closely related to climate, weather, and atmospheric events worldwide. Its forecasting is essential for supporting the decision of governments and environmental organizations. Literature has shown that single machine learning (ML) models are generally more accurate than traditional statistical models for SST time series modeling. However, the parameters tuning of these ML models is a challenging task, mainly when complex phenomena, such as SST forecasting, are addressed. Issues related to misspecification, overfitting, or underfitting of the ML models can lead to underperforming forecasts. This work proposes using hybrid systems (HS) that combine (ML) models using residual forecasting as an alternative to enhance the performance of SST forecasting. In this context, two types of combinations are evaluated using two ML models: support vector regression (SVR) and long short-term memory (LSTM). The experimental evaluation was performed on three datasets from different regions of the Atlantic Ocean using three well-known measures: mean square error (MSE), mean absolute percentage error (MAPE), and mean absolute error (MAE). The best HS based on SVR improved the MSE value for each analyzed series by $82.26\%$, $98.93\%$, and $65.03\%$ compared to its respective single model. The HS employing the LSTM improved $92.15\%$, $98.69\%$, and $32.41\%$ concerning the single LSTM model. Compared to literature approaches, at least one version of HS attained higher accuracy than statistical and ML models in all study cases. In particular, the nonlinear combination of the ML models obtained the best performance among the proposed HS versions.

## Introduction

Climate change is one of humanity’s most critical global challenges since it is harmful to all living beings on Earth. Therefore, it is a crucial subject for all countries, independent of geographical, social, or economic characteristics. Global climate change directly affects the environment and has already had observable effects, such as glacier shrinkage, more intense heat waves, and sea-level rise. According to the Intergovernmental Panel on Climate Change, several regions will be affected in diverse manners over time, leading to significant societal and environmental systems changes. The Sea Surface Temperature (SST) is one of the most significant variables for monitoring the global climate system^1^. SST is related to ocean heat content, which directly affects global warming. The SST records are collected using satellites, which read these values mainly of moored and drifting buoys and can be considered the best-known ocean parameter on global scales^2^.

The variability of the SST is correlated with many natural phenomena^1,3–6^. For instance, the El Niño/Southern Oscillation and Indian Ocean Dipole relate to the warming or cooling of SST in predefined areas of the Pacific and Indian oceans, respectively^1^. Furthermore, the SST from the Atlantic ocean is connected with the quantity of rain and cloudiness in South America, droughts in Northeastern Brazil, and climate change on the Amazon vegetation^4,7^. In this way, SST forecasting can support the decision in many operational applications, such as rainfall monitoring, turtles tracking, tourism, fishing management, and coral bleaching evaluation^8^.

In the time series forecasting literature, statistical and machine learning (ML) models have been widely employed in various domains^9–11^. Among statistical models, linear methods, such as Autoregressive (AR), Moving Average (MA), and ARIMA models are the most popular due to their simplicity, adaptability, and the Box and Jenkins methodology^12^, which provides a well-established design process for time series modeling. The Box & Jenkins methodology used in the design of the linear models guarantees that the linear patterns are properly modeled. However, this class of models is not able to properly model temporal phenomena that present nonlinear patterns. ML models have been highlighted due to their performance, flexibility, nonlinearity and because they are data-driven techniques, allowing temporal modeling without making any a priori assumption^13^. Among the ML models, multilayer perceptron neural networks (MLP), support vector regression (SVR), and long short-term memory (LSTM) are examples of techniques that have reached promising results^11,14,15^.

We can highlight the following works that used linear statistical models and nonlinear ML models for SST forecasting. Lins et al.^3^ employed an SVR to the daily forecast of one year ahead of two different locations of the tropical Atlantic ocean. Salles et al.^4^ applied the ARIMA model to analyze the temporal aggregation of seventeen datasets of SST located in the tropical Atlantic ocean. Tripathi et al.^5^ analyzed the MLP and linear regression techniques in the monthly forecasting of SST located in the Indian Ocean. Mahongo and Deo^1^ showed that the Nonlinear Autoregressive with Exogenous Input neural network reached an accurate performance in the SST forecast located in the African seashore of the Indian ocean. Garcia-Gorriz and Garcia-Sanchez^6^ employed a system based on MLP to the monthly forecasting of the SST in the western Mediterranean Sea.

One of the primary objectives in time series analysis and forecasting is to develop accurate systems. Among the approaches that use ML methods, hybrid systems that combine models from the error series modeling have reached promising results in many applications^16–21^. Residuals or error series are obtained from the difference between the time series and its forecasting. Such hybrid systems use the residuals modeling to correct biased forecasts that can occur due to the overfitting, underfitting, or misspecification of models^22,23^. Hybrid systems commonly design the time series as a combination of a linear statistical model with a nonlinear ML model or as the combination of ML models. The former aims to model the linear and nonlinear patterns of the time series separately^16,17,20^. The latter employs ML models for error series modeling intending to improve the accuracy of an initial nonlinear ML model^19,22,23^.

To the best of our knowledge, hybrid systems that perform the residuals modeling were not proposed or evaluated for SST time series forecasting. In SST forecasting, the works proposed in the literature commonly use a single method to model the time series under analysis^24^. These approaches employ mainly linear statistical models or nonlinear ML models for this task^3–5,24–27^. To fulfill this gap, we perform an empirical evaluation of hybrid systems that use error series modeling in the context of SST time series forecasting. This experimental analysis is of crucial importance because of the adoption of hybrid systems: (i) it generally leads to more accurate results than single models in complex time series modeling^16,17^; (ii) it is an efficient way of dealing with the problem of model selection with little extra effort^19^; and (iii) it is an effective manner to correct biased and/or misspecified forecasters^22,23^. However, the best combination approach between the forecasters of time series and the residuals still is an open question^19^ and not yet investigated in the context of SST forecasting. This work evaluates the performance of hybrid systems combining ML models for SST time series forecasting unprecedentedly. In this sense, the objective of this paper is threefold: (a) evaluate whether the residual modeling is an advantageous approach to increasing ML models’ accuracy for SST time series; (b) evaluate two well-known forms of combination (linear and nonlinear) of the literature employing ML models; (c) analyze, for each SST series, which combination is most suitable.

The main contributions of this work can be summarized as:

- Proposal of a hybrid system methodology to improve the accuracy of ML models in the SST forecasting;
- The performance evaluation of two hybrid systems in the 1-day ahead forecasting SST using three well-known measures: mean square error (MSE), mean absolute percentage error (MAPE) and mean absolute error (MAE);
- The development of two versions of each analyzed hybrid system using well-known ML models: SVR and LSTM;
- The hybrid systems employing the SVR achieved on average a percentage gain compared to its respective single model of 80.27%, 61.72%, and 60.21% for MSE, MAPE, and MAE, respectively;
- The hybrid systems using the LSTM attained an average percentage gain concerning its respective single model of 73.16%, 57.90%, and 56.98% for MSE, MAPE, and MAE, respectively;
- The results show that, in general, the developed hybrid systems overcame literature statistical and ML models in the SST forecasting context.

The remainder of the paper is organized as follows. “Related works” section shows the related works of hybrid systems that deal with residual series modeling. This section describes the evaluated hybrid systems in the SST forecasting: Perturbative approach (“The perturbative approach” section) and NoLiC (“The NoLiC method” section). “PIRATA data set” section presents the data set extracted from the PIRATA project website. “Experimental protocol” section shows the experimental protocol used in this work. In “Simulations and experimental results” section, the results and discussions are presented. Finally, “Discussion” section shows the concluding remarks and suggestions for future works.

## Related works

Combining models is one of the most common alternatives to enhance the accuracy of forecasting systems^16,17,28–31^. In the literature, there are two well-established approaches: ensembles^32^ and hybrid systems that perform residuals modeling^16,17^. Both theoretical and empirical results indicate that the latter approach is an interesting strategy to increase the robustness and accuracy of the forecasts^16,17,28–31^.

The general architecture of a hybrid system that performs the residuals modeling can be divided into three main steps: time series forecasting, error series forecasting, and the combination of the two first steps. Equation (1) shows a general view of this architecture, where the final output of the hybrid system ${\hat{y}}_t$ is given by a function *f*(.) that combines the forecast of the time series $P_0$ with the forecast of the residuals $P_1$ to estimate $y_t$. $P_0$ is the forecast of the time series given by the ${\text {M}}_0$ model (Eq. 2), and $P_1$ is the forecast of the residual series $(e_{t-1},\dots ,e_{t-n})$ given by the ${\text {M}}_1$ model (Eq. 3). The residual series is calculated as the difference between the predicted and the actual values.

1 $$\begin{aligned} {\hat{y}}_t = f(P_0,P_1), \end{aligned}$$

where

2 $$\begin{aligned} P_0 = {\mathrm{M}}_0(y_{t-1},\dots ,y_{t-m}), \end{aligned}$$

and

3 $$\begin{aligned} P_1 = {\mathrm{M}}_1(e_{t-1},\dots ,e_{t-n}), \end{aligned}$$

where *m* and *n* are the time lags used as input to the ${\text {M}}_0$ and ${\text {M}}_1$ models, respectively. The time lags can be defined using the auto-correlation function (ACF), partial auto-correlation function (PACF), or some searching algorithm^31,33^.

Based on the general architecture described by the *f* function in Eq. (1), two classes of hybrid systems have been studied for real-world time series modeling: a combination of linear statistical methods with nonlinear Machine Learning (ML) models and a combination of ML models. For simplicity, the first class is denominated as a hybrid system and the second as a combination of ML models. The hybrid system class is described in “Hybrid systems—combining linear and nonlinear models” section. Techniques that combine ML models are presented in “Combining nonlinear models” section. This section also describes two recent techniques: the perturbative approach^22^ and NoLiC^23^.

### Hybrid systems—combining linear and nonlinear models

Linear statistical models have been combined with nonlinear ML models based on the assumption that real-world time series generally present linear and nonlinear patterns^16,17^. Thus, in this hybrid system class, statistical models are used as ${\text {M}}_0$, and ML models are employed as ${\text {M}}_1$ intended to deal with linear and nonlinear patterns separately.

The *f* function that is responsible for combining $P_0$ with $P_1$ can be either linear or nonlinear^16,19,23,34^. The linear combination, which is more commonly used in the literature^16,29,35^, consists of a non-trainable rule, such as the sum. This combination has been successfully used in several applications, for instance: financial indexes^29^, wind speed^35^, groundwater level fluctuations^36^, the prevalence of schistosomiasis in humans^37^, particulate matter^38^, and water quality^39^.

Zhang^16^ showed the linear combination:

4 $$\begin{aligned} {\hat{y}}_t = P_0 + P_1, \end{aligned}$$

where $P_0$ is the forecasting of a linear statistical model (${\text {M}}_0$), $P_1$ is the forecasting of an ML model (${\text {M}}_1$) applied to the residual of the time series, and ${\hat{y}}_t$ is the final prediction of the hybrid system performed by the linear combination. In his experiments, $M_0$ was defined as an ARIMA and $M_1$ as an MLP neural network.

Despite being widely used in the literature, the linear combination of the forecasts $P_0$ and $P_1$ can underestimate, or degenerate the accuracy of the initial model (${\text {M}}_0$), since there may be no additive relationship between linear and nonlinear forecasts^17,40–42^.

Based on this assumption, Khashei and Bijari^17,41,42^ proposed a nonlinear combination of the forecasts to overcome the limitations of the linear combination. In their hybrid systems^17,41,42^, the function *f* (Eq. 1) is defined as an ML that receives as input $P_0$, the residual ($(e_{t-1},\dots ,e_{t-n})$), and the time series ($y_{t-1},\dots ,y_{t-m}$), as shown in Eq. (5). They employed an ARIMA model for time series modeling as $P_0$, and an MLP as $P_1$.

5 $$\begin{aligned} {\hat{y}}_t = f(P_0, e_{t-1},\dots ,e_{t-n},y_{t-1},\dots ,y_{t-m}). \end{aligned}$$

In general, the nonlinear combination of the forecasts $P_0$ and $P_1$ reaches better results than the linear approach^17,41,43^. However, there is no guarantee that the nonlinear combination is the most appropriate for modeling any temporal phenomena^42^. Therefore, the best combination function of the forecasts of the time series (linear component) and the residual series (nonlinear component) is unknown, being still a research challenge in the hybrid systems research field^17,23,42^.

### Combining nonlinear models

Nonlinear ML models have been combined based on the assumption that adopting only one single model can be inadequate to real-world time series forecasting. The underperforming of a single ML model can occur due to problems caused by overfitting, underfitting, or misspecification^22,23,28^.

In Ginzburg and Horn^28^, two MLPs are combined linearly following the same idea shown in Eq. (4). Thus, the time series forecast ($P_0$) is performed by the first MLP (${\text {M}}_0$), and its residuals are modeled by the second MLP (${\text {M}}_1$), generating the forecast of the residuals ($P_1$). In this sense, the ${\text {M}}_1$ model is employed to uncover and model temporal correlations found in the residuals of ${\text {M}}_0$, thus correcting the original forecast ($P_0$). This premise is based on biological systems that commonly deal with complex tasks through subsystems^28^. Later stages of consecutive subsystems (networks) refine the response of earlier ones, improving the performance of the entire biological system^28,44^. This principle was also successfully employed in atmospheric pollution forecasting^31,45^.

The next two subsections show two recent approaches that combine nonlinear models: the perturbative approach^22^ and NoLiC^23^.

#### The perturbative approach

The linear combination of ML models proposed in^28^ was generalized in^22^, which employed the perturbation theory concept that was previously applied in many areas, such as physics, chemistry, and mathematics^46,47^. The idea is to initiate the forecasting of a time series using a first estimation (forecast $P_0$). Then, *p* new forecasts ($P_1 + P_2 + \cdots + P_p$) are added to make a partial forecast ever closer to the real solution *P*. Mathematically,

$$\begin{aligned} P = P_0 + P_1 + P_2 + \cdots + P_p, \end{aligned}$$

where *P* is the desired solution (perfect forecasting), $P_0$ is the series forecast, and the term of the major contribution to *P*, and $P_1, P_2, \dots , P_p$ are the *p* higher-order terms (residual forecasts). Then, $P_1$ is the forecast of the residuals of ${\text {M}}_0$, $P_2$ is the forecast of the residuals of ${\text {M}}_0$ $+$ ${\text {M}}_1$, $P_3$ is the forecast of the residuals of ${\text {M}}_0$ $+$ ${\text {M}}_1+{\text {M}}_2$, and $P_p$ is the forecast of the residuals of ${\text {M}}_0+{\text {M}}_1+{\text {M}}_2+\cdots +{\text {M}}_{p-1}$. Theoretically, the corrections generated by the residual forecasts ($P_1, P_2, \dots , P_p$) decrease since, at each perturbation *i*, the residual series $E_i$ present values closer to zero. In practice, the contribution of the residual forecasts ($P_1, P_2, \dots , P_p$) depends on the specification and training of the model $M_i$.

Algorithms 1 and 2 show the training and the testing phases of the perturbative approach^22^, respectively.

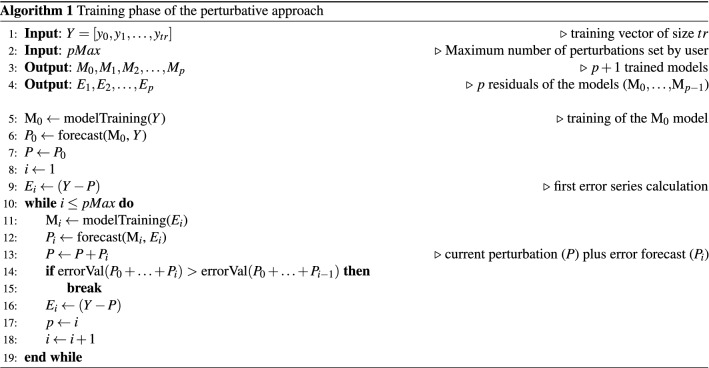



The training phase is divided into two general steps: training the time series forecasting model (lines 5–9) and training the correction models based on the residual series (lines 10–19). The algorithm’s input is the training set of the time series, and the outputs are *p* residuals (error series *E*) and $p+1$ trained models ($\{M_0, M_1, \ldots , M_p\}$). The training phase has two stop criteria: the maximum number of perturbations (*pMax*) or an increase in the error value in the validation set concerning antecedent perturbation (lines 14 to 15).

In line 4, the initial model (${\text {M}}_0$) is trained using the training set *Y*, generating the time series forecast $P_0$ in line 5. $P_0$ is the main contributor to the final solution *P*^22^. In line 8, the first error series ($E_1$) is generated. The error series consists of the difference between the actual series and the estimated values provided by the perturbative approach (*P*).

After, the perturbative terms are generated. Each ${\text {M}}_i$ model is trained to forecast $E_{i}$ (line 10), which is the difference between *Y* and *P* (line 14). At each iteration of the loop (lines 9–15), a new perturbative term is generated (lines 10–11) and added to the final solution (line 12). At the end of the training phase, *P* is the sum of the $p+1$ forecasts ($P_0, P_1, P_2, \dots , P_p$) of the $p+1$ models (${\text {M}}_0+{\text {M}}_1+{\text {M}}_2 \dots + {\text {M}}_p$). Lines 10 to 14 are executed until the stopping criterion is reached.

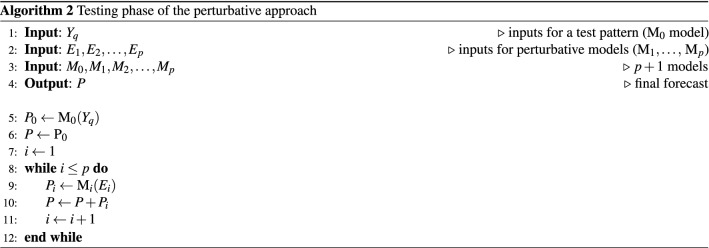



The testing phase (Algorithm 2) is divided into two steps: forecast of the time series (line 5) and forecast of the perturbative terms (lines 8–12). Lines 5 and 6 show the generating of $P_0$ and its inclusion in the final output *P* of the perturbative approach. Lines 8 to 12 show the second part, where the perturbative terms are generated for the test point (observation of the test sample). So, each model (${\text {M}}_1$, ${\text {M}}_2, \dots , {\text {M}}_p$) generate the forecasting of its respective error series (${\text {E}}_1, {\text {E}}_2, \dots , {\text {E}}_p$). This loop is repeated (*p*) times, which is the number of perturbations defined in the training phase. After, each perturbative term is forecasted (line 9) and added to the solution *P* using a linear combination (line 10), generating the final forecasting.

#### The NoLiC method

The NoLiC method^23^ employs an adaptive combination of ML models with other techniques using the residual series. This combination method does not presuppose a linear combination as other works^16,22,28^. The idea is to find a combination function between $P_0$ and $P_1$ using an ML model that is flexible, capable of performing linear and nonlinear modeling.

**Figure 1:**
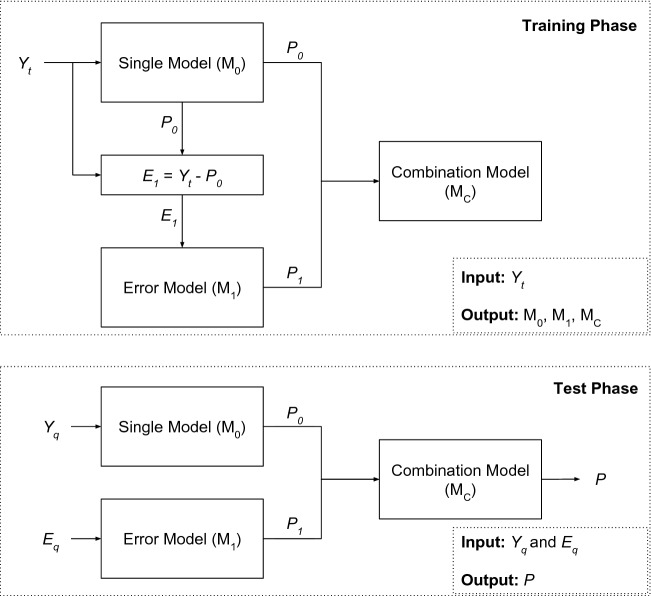
NoLiC training and testing phases.

The Nonlinear Combination (NoLiC) method is composed of three steps: forecast of the time series ($P_0$), forecast of the residuals ($P_1$), and the combination *f*(.) of $P_0$ and $P_1$. Figure 1 shows the training and testing phases of the NoLiC method.

The training phase receives as input the training set and generates three trained models (${\text {M}}_0$, ${\text {M}}_1$, and M$_{\mathrm{C}}$) as outputs. Similarly to other works^16,28^, the models ${\text {M}}_0$ and ${\text {M}}_1$ are employed to forecast the time series and the residuals, respectively. The ${\text {M}}_1$ model’s training is performed using the residuals of ${\text {M}}_0$ ($E_1 = Y_t - P_0$), generating the forecast of the error series $P_1$. After, the combination model M$_{\mathrm{C}}$ receives as inputs $P_0$ and $P_1$ and is trained with the objective to correct the output of ${\text {M}}_0$, generating a forecast (*P*) closer to the target (future value of $Y_t$).

In the test phase, the ${\text {M}}_0$ and ${\text {M}}_1$ models receive the lag values of the time series ($Y_q$) and the residuals ($E_q$), respectively. After, ${\text {M}}_0$ and ${\text {M}}_1$ models generate theirs respective forecasts $P_0$ and $P_1$. Then, the trained ML model ${\text {M}}_C$ combines the forecasts of the series and residuals to produce the final forecast *P*.

#### Remarks

The combination methods described in “The perturbative approach” and “The NoLiC method” sections have different characteristics. The perturbative method can extract information from more than one residual series. However, this method supposes that the models should be combined using a simple sum rule. In contrast, the NoLiC method supposes a nonlinear combination between the forecasts and the residuals.

The NoLiC method employs an ML model aiming to find a combination more suitable than a simple sum. However, there is no guarantee that the ${\mathrm{M}}_{\mathrm{C}}$ model leads to the best accuracy of the hybrid system. The optimum performance depends on adjusting the parameters and training of the ${\mathrm{M}}_{\mathrm{C}}$ model, which is a complex task since it is related to forecasts of ${\mathrm{M}}_0$ and ${\mathrm{M}}_1$. Thus, investigating how to combine the forecasts of the time series and its error series is a crucial issue in the definition of the hybrid system since it is closely related to its accuracy.

## PIRATA data set

**Figure 2:**
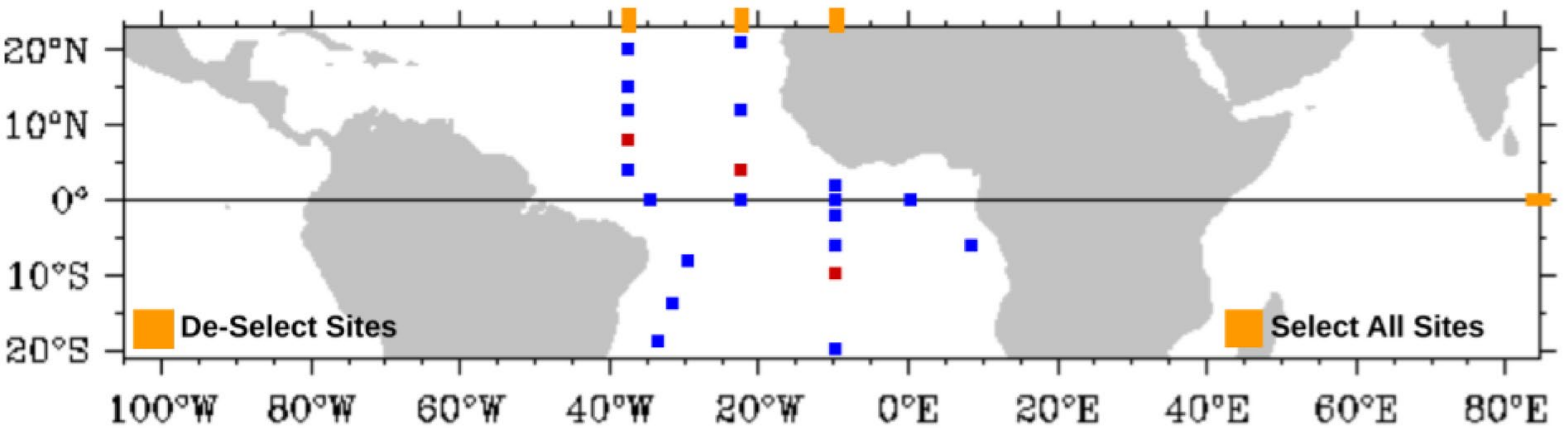
Buoys locations of the PIRATA project in the Atlantic Ocean. Font: https://www.pmel.noaa.gov/tao/drupal/disdel/.

The Pilot Research Moored Array in the Tropical Atlantic (PIRATA) was developed by a network of observatories composed of many countries, such as Brazil, France, and the United States. This project has the objective to improve the knowledge about atmospheric variations in the tropical Atlantic Ocean^48^. The climatic variations in this area can influence the development of droughts, floods, severe storms, and even hurricanes, affecting millions of people in South America and Africa^3^ (Fig. 2).

The project PIRATA has buoys in the ocean, where meteorological variables are collected, such as shortwave radiation, relative humidity, air temperature, and ocean surface temperature. All data are gathered and transmitted by satellite and are available on the project web page (https://www.pmel.noaa.gov/gtmba/pirata).

**Figure 3:**
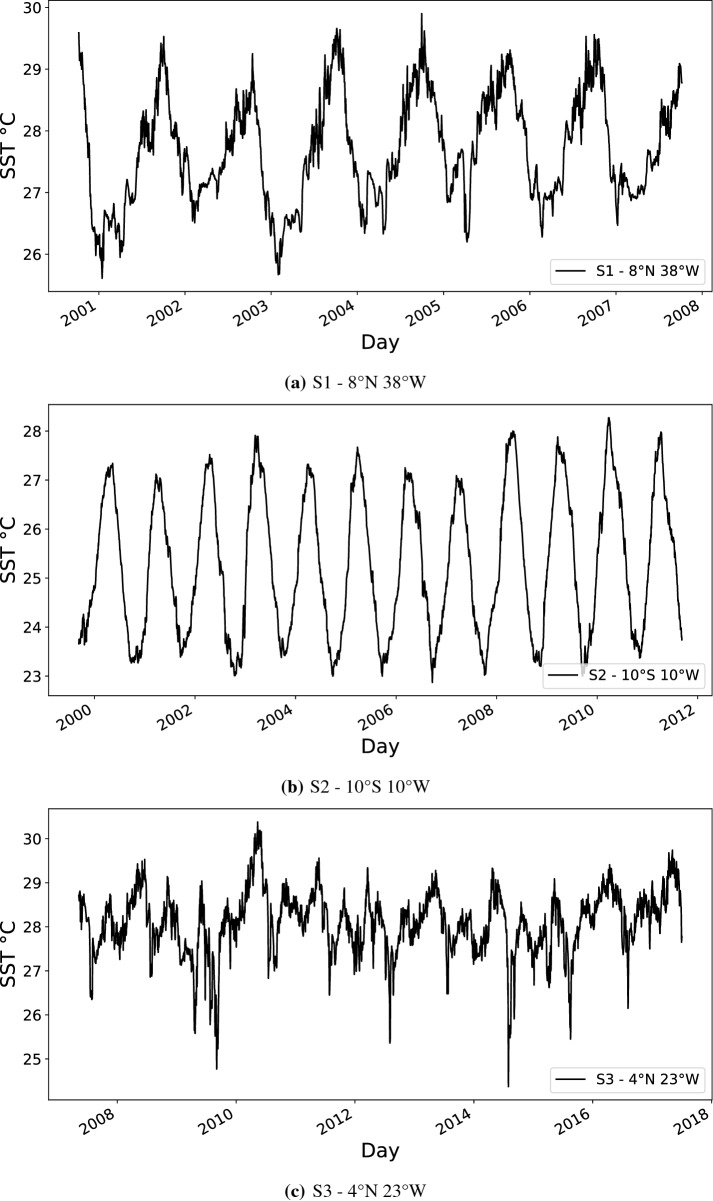
Sea surface temperature time series.

We aim to perform the forecast of the sea surface temperature of three regions^3^: $8^\circ$ N $38^\circ$ W (Fig. 3a), $10^\circ$ S $10^\circ$ W (Fig. 3b), and $4^\circ$ N $23^\circ$ W (Fig. 3c). These locations were selected because they have an appropriate amount of data for modeling with ML models. These data sets do not present interruptions and are located in different regions. The selected locations can be seen in Fig. 2, represented by red points. Table 1 shows the characteristics of each time series used in this work.

**Table 1:** Properties of the SST time series used in this work.

Adopted acronym	S1	S2	S3
**Time series**			
Localization	$8^\circ$ N $38^\circ$ W	$10^\circ$ S $10^\circ$ W	$4^\circ$ N $23^\circ$ W
Time unit	Daily	Daily	Daily
Start date	Oct 08, 00	Sep 09, 99	May 10, 07
Start date (test set)	Oct 01, 06	Sep 07, 10	Jun 30, 16
End date	Oct 07, 07	Sep 08, 11	Jul 02, 17
Total size	2545	4376	3699
Training sample size	1815	3646	2969
Validation sample size	365	365	365
Testing sample size	365	365	365

## Experimental protocol

The experiments evaluate two machine learning models: support vector machines (SVR) and long short-term memory (LSTM). These models were chosen because they reached relevant results regarding accuracy for the SST forecasting task^3,49,50^. The SVR and LSTM models are employed as single models as well as in the combination approaches.

The SVR model was successfully employed in the SST forecasting^3^ and has highlighted results in several other forecasting applications^51^. SVR is an interesting choice because it employs a quadratic optimization procedure to solve a convex constrained problem, with a single solution^52^. Therefore, in contrast to methods such as neural networks where several local minima can be achieved, the uniqueness of the solution of SVR is obtained given a set of hyperparameters. To the kernel SVR, the Radial Basis Function (RBF) was selected because it is a well-established kernel function in the time series forecasting area^51^. RBF kernel also was successfully employed in the SST forecasting^3^ and has been widely used in hybrid systems^18–20,29,53^. Besides, the RBF is considered the default SVR kernel in the Sklearn^54^ library, which is now the most popular package for creating SVR models in Python. The RBF’s popularity can be explained by its finite and localized responses across the entire range of the x-axis, so it does not need previous assumptions about the data and adds few parameters to the SVR model (Cost and Gamma)^55^.

LSTM was selected because it is one of the state-of-the-art ML models in time series forecasting. It has outperformed traditional neural networks in several applications^56^. Its ability to deal with short or long-term temporal dependencies can be promising in the SST time series modeling^57^.

For the combination approach, the same model (SVR or LSTM) are employed for all stages. For the perturbative approach, the best number of perturbations (or corrections) was selected based on the MSE value in the validation set having a upper limit of four perturbations. For all models, a grid search approach was performed for selecting the best configuration based on the MSE value in the validation set. The data used in the experimental simulations were scaled into the interval [0.1, 0.9], similar to^3^.

Table 2 shows the set of parameters investigated for each model in the 1-day-ahead forecasting scenario. The number of input lags used in the grid search was selected based on PACF. For S1, the lags 1, 3, 4, 11, and 15 presented significant linear correlations. For S2, PACF selected the lags 1, 2, 3, 4, 5, 6, 7, 8, 9, 10, 11, 13, 15, 16, 17, 18, 19, 20, 21, 22, 23, 24, 28, and 30, and for S3, the lags 1, 2, 3, 5, 17, and 18 presented relevant correlation.

**Table 2:** Values of the parameters of the SVR and LSTM models.

Model	Parameters	Values
SVR	Gamma	[0.001, 1]
	Cost	[0.1, 1, 100]
	Tolerance	[0.001, 0.01, 0.1]
	Kernel	Radial basis function^3^
LSTM	Units in hidden layer	[2, 5, 10]
	Algorithm	Adam^19,58^

Table 3 shows the values selected for single and combination approaches for 1 day ahead SST forecasting for each study case (S1, S2, and S3 series). It is important to highlight that all combination approaches use the same ${\text {M}}_0$, and the NoLiC method employs the same ${\text {M}}_0$ and ${\text {M}}_1$ of the perturbative approach. So, it is possible to compare the performance of the combination approaches directly. For all series, the perturbation approach employed three perturbations for the SVR model and two perturbations for the LSTM model.

**Table 3:** Selected parameters for SVR and LSTM in the combination approaches using a grid search in the validation set for 1 day ahead SST forecasting.

Time series	Model	Parameters	Combination approaches				
			Perturbative^22^				NoLiC^23^
			${\mathrm{M}}_0$	${\mathrm{M}}_1$	${\mathrm{M}}_2$	${\mathrm{M}}_3$	${\mathrm{M}}_{\mathrm{C}}$
S1	SVR	Gamma	1	1	0.001	1	1
		Cost	1	0.1	1	1	100
		Tolerance	0.001	0.001	0.01	0.001	0.01
		Inputs	2	2	1	1	2
	LSTM	Units in hidden layer	2	5	5	–	10
		Inputs	1	2	2	–	2
S2	SVR	Gamma	0.001	1	1	0.001	1
		Cost	100	1	0.1	100	1
		Tolerance	0.001	0.01	0.01	0.001	0.001
		Inputs	23	2	2	2	2
	LSTM	Units in hidden layer	5	10	5	–	5
		Inputs	5	2	2	–	2
S3	SVR	Gamma	1	0.001	1	1	1
		Cost	1	100	1	100	100
		Tolerance	0.001	0.01	0.01	0.01	0.01
		Inputs	3	2	2	2	2
	LSTM	Units in hidden layer	5	5	5	–	10
		Inputs	5	2	2	–	2

The performance of the approaches are evaluated using three performance metrics applied to the context of sea surface temperature forecast^3,4^: mean square error (MSE), mean absolute percentage error (MAPE) and mean absolute error (MAE). Equations (6), (7) and (8) show the MSE, MAPE and MAE metrics, respectively.

6 $$\begin{aligned} {\mathrm{MSE}}= & {} \displaystyle \frac{1}{N}\sum _{t=1}^{N}(y_t - {\hat{y}}_t)^2, \end{aligned}$$

7 $$\begin{aligned} {\mathrm{MAPE}}= & {} \displaystyle \frac{100}{N}\sum _{t=1}^{N} \left| \frac{y_t - {\hat{y}}_t}{y_t}\right| , \end{aligned}$$

8 $$\begin{aligned} {\mathrm{MAE}}= & {} \displaystyle \frac{1}{N}\sum _{t=1}^{N} |y_t - {\hat{y}}_t|, \end{aligned}$$

where *N* represents the time series length, $y_t$ the true value at time *t* and ${\hat{y}}_t$ is the forecast at time *t*. For all metrics, the lower the values, the better the results. A percentage gain/loss measure (Eq. 9) is used to compare the combination approaches with the single models.

9 $$\begin{aligned} {\mathrm{PC}} = \displaystyle \frac{{\mathrm{Metric}}_{\mathrm{sm}} - {\mathrm{Metric}}_{\mathrm{comb}}}{{\mathrm{Metric}}_{\mathrm{sm}}}\times 100, \end{aligned}$$

where ${\mathrm{Metric}}_{\mathrm{sm}}$ and ${\mathrm{Metric}}_{\mathrm{comb}}$ represent the MSE values reached by single models and combination approaches, respectively. In this way, the higher the PC, the better the performance of the combination approach in relation to the single model.

The SVR and LSTM models were implemented in the Python programming language using the Sklearn^54^ and Keras^59^ libraries. The experimental simulations were performed in a computer with a single Intel Core i7-7500 CPU and 20 GB RAM.

The experimental comparison was carried out among the single models, the hybrid approaches, and the following literature models: Exponential Smoothing (ETS)^60^, Convolution LSTM (ConvLSTM)^11,50^, and the Nonlinear Autoregressive Exogenous (NARX)^1^.

Exponential Smoothing (ETS) is a traditional statistical method employed in time series forecasting^60,61^. It is a versatile method due to its ability to model time series with/without trend and seasonality components. However, the ETS can reach a limited performance in forecasting time series that present nonlinear patterns^62^. The experiments with ETS were carried out using the Statsmodel library of Python^63^.

The Convolutional Long Short-Term Memory (ConvLSTM)^11,50^ is a Deep Learning technique able to model spatiotemporal correlations. The ConvLSTM models spatial and temporal patterns using convolution and LSTM layers. This technique attained higher accuracy than other ML models to SST time series forecasting tasks^11,50^. On the other hand, its training can be costly computationally due to the number of hyper-parameters that must be adjusted^64^. In this work, the employed ConvLSTM used the configuration suggested in the SST forecasting works^11,50^.

Nonlinear Autoregressive with Exogenous Input neural network (NARX) was proposed to model the nonlinear and autoregressive behaviors^65^. NARX model was successfully used to predict SST anomalies in the western Indian Ocean region^1^. Despite being able to forecast seasonal anomaly trends, the NARX performance is highly sensitive to parameters specification^1^.

## Simulations and experimental results

Table 4 shows the results regarding MSE, MAPE and MAE for the test set of the S1, S2, and S3 series, for 1-day ahead forecasting. In that table is possible to compare the performance of the hybrid systems with single and literature models.

**Table 4:** Comparison in terms of MSE, MAPE, and MAE of the combination approaches with single statistical and Machine Learning models of the literature applied to the SST daily forecasting.

Dataset	Approach	Model	MSE	MAPE	MAE
**S1**	Perturbative	SVR	6.89E−04	3.61	1.85E−02
		LSTM	6.89E−04	3.61	1.85E−02
	NoLiC	SVR	6.71E−04	3.60	1.84E−02
		LSTM	**3.97E−04**	**2**.**65**	**1.38E−02**
	Literature	ETS^60^	5.36E−03	12.30	5.85E−02
		Single SVR^3,19^	3.78E−03	9.40	4.56E−02
		Single LSTM^19,58^	5.06E−03	10.83	5.21E−02
		ConvLSTM^11,50^	1.39E−03	5.53	2.72E−02
		NARX^1^	8.77E−04	5.28	2.51E−02
**S2**	Perturbative	SVR	**1.08E−04**	**1.97**	**7.82E−03**
		LSTM	**1.08E−04**	1.98	7.86E−03
	NoLiC	SVR	1.01E−03	4.52	2.08E−02
		LSTM	1.31E−04	2.19	8.82E−03
	Literature	ETS^60^	3.87E−03	13.93	5.43E−02
		Single SVR^3,19^	1.01E−02	20.83	8.78E−02
		Single LSTM^19,58^	8.30E−03	18.86	7.93E−02
		ConvLSTM^11,50^	8.59E−04	5.92	2.33E−02
		NARX^1^	2.03E−04	3.04	1.16E−02
**S3**	Perturbative	SVR	9.38E−04	3.80	2.36E−02
		LSTM	7.91E−04	**3.41**	**2.11E−02**
	NoLiC	SVR	9.02E−04	3.75	2.34E−02
		LSTM	**7.74E−04**	3.43	2.15E−02
	Literature	ETS^60^	5.78E−03	9.00	5.56E−02
		Single SVR^3,19^	2.58E−03	6.20	3.75E−02
		Single LSTM^19,58^	1.15E−03	3.96	2.44E−02
		ConvLSTM^11,50^	1.27E−03	4.30	2.60E−02
		NARX^1^	9.18E−04	3.89	2.35E−02

For each data set, the best value of the metrics is highlighted in bold.

For the S1 time series, all hybrid systems improved the accuracy of their respective single model, reaching better MSE, MAPE, and MAE values better than literature models. In particular, the NoLiC employing the LSTM model attained the best result in all considered metrics. Regarding MSE, the hybrid system versions obtained an error of one order of magnitude smaller than their respective single models, for instance, 3.97E−04 for NoLiC+LSTM and 6.89E−04 for LSTM.

The S2 and S3 series follow the same behavior: all hybrid systems versions improved the performance of their respective single models for the evaluated metrics. For the S2 time series, the perturbation approach using the SVR model attained the best performance in terms of MSE, MAPE, and MAE. This hybrid system version, which employed three perturbations ($P_0 + P_1 + P_2 + P_3$), improved the MSE value in two orders of magnitude regarding the single SVR.

Hybrid systems that use the LSTM model deserve special attention for the S3 time series. In this case, the NoLiC attained the best MSE value, while the perturbative approach ($P_0 + P_1 + P_2$) obtained the smallest MAPE and MAE. Both hybrid system versions of single SVR and LSTM improved the MSE value in one order of magnitude. Among the single and literature models, the NARX^1^ achieved the best results for the evaluated times series.

**Figure 4:**
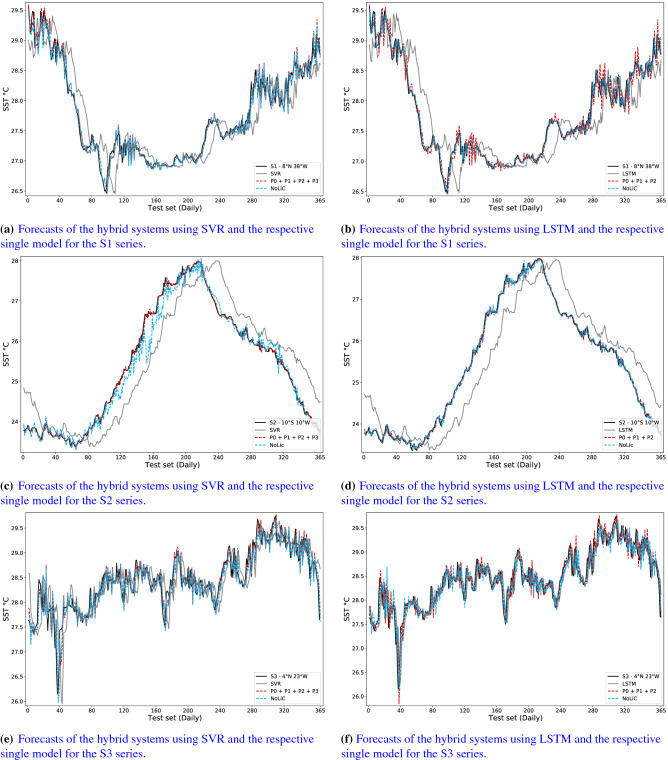
One day ahead forecasting for the SST time series on the test set with Perturbative approach, NoLiC and the respective single model.

Tables 5 and 6 show the percentage difference (Eq. 9) in terms of the MSE metric between the literature models and the perturbative and NoLiC approaches, respectively. The tables show that the hybrid systems improved the performance of both single models for the S1 series. The NoLiC using the LSTM model attained an improvement greater than 65% for all evaluation metrics (Table 5). The versions of the hybrid systems attained a superior performance, at least 20% when compared with the literature. Figure 4a,b show the forecasts of the S1 series test set of the hybrid approaches using SVR and LSTM, respectively. It can be seen that both hybrid systems were able to improve the forecasting of the single models. Both hybrid approaches achieved forecasts closer to the real when compared with the initial model.

**Table 5:** Percentage difference between the perturbative approach and literature models for MSE, MAPE, and MAE.

Dataset	Model	Pertubative approach					
		SVR			LSTM		
		MSE	MAPE	MAE	MSE	MAPE	MAE
S1	ETS^60^	87.14	70.63	68.27	87.14	70.63	68.27
	Single SVR^3,19^	81.78	61.59	59.29	81.78	61.59	59.29
	Single LSTM^19,58^	86.37	66.65	64.41	86.37	66.65	64.41
	ConvLSTM^11,50^	50.25	34.73	31.85	50.25	34.73	31.85
	NARX^1^	21.44	31.63	26.15	21.44	31.63	26.15
S2	ETS^60^	97.20	85.88	85.59	97.20	85.79	85.51
	Single SVR^3,19^	98.93	90.56	91.09	98.92	90.50	91.05
	Single LSTM^19,58^	98.70	89.57	90.13	98.69	89.51	90.08
	ConvLSTM^11,50^	87.39	66.75	66.40	87.38	66.55	66.21
	NARX^1^	46.76	35.27	32.71	46.71	34.88	32.34
S3	ETS^60^	83.76	57.73	57.62	86.31	62.14	61.98
	Single SVR^3,19^	63.64	38.66	37.20	69.35	45.06	43.65
	Single LSTM^19,58^	18.07	3.94	3.36	30.93	13.97	13.30
	ConvLSTM^11,50^	25.90	11.48	9.25	37.54	20.72	18.57
	NARX^1^	− 2.15	2.16	− 0.30	13.89	12.37	10.01

**Table 6:** Percentage difference between the NoLic and literature models for MSE, MAPE, and MAE.

Dataset	Model	NoLiC					
		SVR			LSTM		
		MSE	MAPE	MAE	MSE	MAPE	MAE
S1	ETS^60^	87.48	70.76	68.58	92.59	78.46	76.36
	Single SVR^3,19^	82.26	61.75	59.69	89.51	71.83	69.67
	Single LSTM^19,58^	86.73	66.79	64.75	92.15	75.54	73.48
	ConvLSTM^11,50^	51.57	35.00	32.51	71.35	52.13	49.23
	NARX^1^	23.51	31.91	26.87	54.75	49.86	44.98
S2	ETS^60^	73.98	67.52	61.60	96.62	84.29	83.74
	Single SVR^3,19^	90.01	78.28	76.28	98.70	89.50	89.95
	Single LSTM^19,58^	87.87	76.01	73.72	98.42	88.40	88.87
	ConvLSTM^11,50^	− 17.24	23.53	10.49	84.76	63.02	62.09
	NARX^1^	− 395.08	− 48.86	− 79.26	35.64	28.00	24.09
S3	ETS^60^	84.38	58.30	57.94	86.60	61.86	61.29
	Single SVR^3,19^	65.03	39.50	37.68	70.00	44.66	42.64
	Single LSTM^19,58^	21.20	5.25	4.10	32.41	13.34	11.74
	ConvLSTM^11,50^	28.73	12.69	9.94	38.87	20.14	17.11
	NARX^1^	1.75	3.50	0.47	15.73	11.73	8.39

For S2, Table 5 shows that the perturbative approach reached an improvement higher than 30% in all comparisons. This approach with SVR obtained a percentage gain regarding SVR of 98.93%, 90.56%, and 91.09% for MSE, MAPE, and MAE, respectively. Table 6 shows the NoLiC using LSTM model attained a gain concerning to single LSTM of 98.42%, 88.40% and 88.87% for MSE, MAPE, and MAE, respectively. Figure 4c,d show the forecasts of the S2 series test set of the hybrid approaches using SVR and LSTM, respectively. Both figures show that the hybrid systems improved the forecast of the single models. In both comparisons, it is possible to verify that the forecast of the hybrid systems using SVR or LSTM is closer to the test set of S2 than the respective single model.

Tables 5 and 6 show that the percentage difference between hybrid systems with LSTM and single models is positive in all comparisons for the S3 data set. The NoLiC using SVR obtained the greatest improvement regarding single SVR with 65.03%, 39.50%, and 37.68% for MSE, MAPE, and MAE, respectively. Figure 4e,f show the forecasts of the S2 series test set of the hybrid approaches using SVR and LSTM, respectively. The forecasts obtained by the perturbative and NoLiC approaches are closer to S3 series when compared with the single models. Supplementary Information presents additional analyzes.

### Discussion

To verify if there are (or not) significant statistical differences between the hybrid systems and literature approaches, we employed the Diebold–Mariano statistical test^66^. We use MSE since it is the target metric employed to guide the search of the parameters of the models. Table 7 shows that both versions of the perturbative approach attain MSE values statistically different from the single and literature models, i.e., the *p* value is smaller than the significance level adopted (0.05) in all comparisons. The NoLiC employing LSTM also reached results statistically better than other models. Only the NoLiC version using SVR attained an MSE worse than NARX^1^ and ConvLSTM^11,50^ models.

**Table 7:** Results of the comparison of the hybrid systems using SVR and LSTM with single and literature models using Diebold–Mariano hypothesis test.

Dataset	Model	Perturbative		NoLiC	
		SVR	LSTM	SVR	LSTM
S1	ETS^60^	+	+	+	+
	Single SVR	+	+	+	+
	Single LSTM	+	+	+	+
	NARX^1^	+	+	+	+
	ConvLSTM^11,50^	+	+	+	+
S2	ETS^60^	+	+	+	+
	Single SVR	+	+	+	+
	Single LSTM	+	+	+	+
	NARX^1^	+	+	−	+
	ConvLSTM^11,50^	+	+	−	+
S3	ETS^60^	+	+	+	+
	Single SVR	+	+	+	+
	Single LSTM	+	+	+	+
	NARX^1^	+	+	+	+
	ConvLSTM^11,50^	+	+	+	+

Table 8 shows the execution time (in seconds) of the testing phase calculated over 30 executions. The evaluated approaches presented an execution time smaller than 1 s in all data sets. It is important to highlight that the hybrid systems based on the LSTM model are more costly regarding computational effort than the ones based on SVR. For instance, the SVR’s perturbative approaches were less computationally costly than single LSTM for S1 and S2 series.

**Table 8:** Testing time in seconds of the single models and combination approaches for 1 day ahead forecasting.

Datasets	Model	Approach	Execution time
			Mean (Std)
S1	SVR	Single	0.014 (0.002)
		${\text {P}}_{{0}} + {\text {P}}_{{1}} + {\text {P}}_{{2}} + {\text {P}}_{{3}}$	0.053 (0.005)
		NoLiC	0.064 (0.120)
	LSTM	Single	0.117 (0.037)
		${\text {P}}_{{0}} + {\text {P}}_{{1}} + {\text {P}}_{{2}}$	0.371 (0.088)
		NoLiC	0.261 (0.057)
S2	SVR	Single	0.011 (0.005)
		${\text {P}}_{{0}} + {\text {P}}_{{1}} + {\text {P}}_{{2}} + {\text {P}}_{{3}}$	0.062 (0.011)
		NoLiC	0.303 (0.027)
	LSTM	Single	0.105 (0.009)
		${\text {P}}_{{0}} + {\text {P}}_{{1}} + {\text {P}}_{{2}}$	0.371 (0.036)
		NoLiC	0.271 (0.016)
S3	SVR	Single	0.020 (0.005)
		${\text {P}}_{{0}} + {\text {P}}_{{1}} + {\text {P}}_{{2}} + {\text {P}}_{{3}}$	0.100 (0.012)
		NoLiC	0.282 (0.024)
	LSTM	Single	0.098 (0.025)
		${\text {P}}_{{0}} + {\text {P}}_{{1}} + {\text {P}}_{{2}}$	0.421 (0.107)
		NoLiC	0.338 (0.043)

For each approach is presented the mean testing time and the respective standard deviation.

The complexity analysis of the hybrid system can be divided into *p* steps, each one corresponding to the training of a model (${\text {M}}_0, {\text {M}}_1, \ldots , {\text {M}}_p$). The evaluated hybrid systems are trained sequentially, and their training time can be described as ${\text {MT}}_0 + {\text {MT}}_1 + \cdots + {\text {MT}}_p$, where MT is the training time of the model in a specific phase. In this way, the NoLiC approach is approximately three times more expensive than the single models, because the NoLiC uses three models (${\text {M}}_0, {\text {M}}_1$, and $M_{\mathrm{C}}$), and the perturbative approach is approximately *p* times more expensive than the single models because it uses ${\text {M}}_0, {\text {M}}_1, \ldots , {\text {M}}_p$ models. This work applies two compositions of hybrid systems, using SVR or LSTM. The SVR training process has a complexity of *O*(*lm*)^18,67^, where *l* is the size of the data set, and *m* represents the number of input features. The training process of the LSTM has a complexity of O(*W*), where *W* is the total number of parameters^68^.

## Conclusion

The Sea Surface Temperature (SST) is an important environmental variable due to its strong relationship to climate, weather, and nature events, such as El Niño. So, the SST accurate forecast can support decisions in several science fields.

In this work, we evaluated two types of hybrid systems intending to improve the performance of single ML models in the task of SST forecast. The hybrid systems are evaluated in the 1-day-ahead forecasting scenario. The purpose was to correct biased and deteriorated forecasts of the ML models by modeling the error series. The Perturbative and NoLiC hybrid approaches employ linear and nonlinear combinations, respectively. For each approach, two versions were generated, one using SVR and another using LSTM as base models. All the models were evaluated in three data sets of different locations in the tropical Atlantic using traditional metrics (MSE, MAPE, and MAE) of the literature. Compared with the ML single models, the hybrid system approaches obtained a significant performance improvement (more than 20%).

Regarding the hybrid systems, it was possible to verify the influence of the combination function in their performance. The linear combination used by the Perturbative approach obtained the best performance in two out of three study cases regarding MSE. Although NoLiC employs a combination function more versatile than the simple sum, it could not overcome the linear combination in most cases. In particular, when the perturbative approach uses the LSTM as the base model, it reached the highest performance in three out of five cases. The LSTM’s best performance compared to the other ML models can be attributed to its ability to capture long-term temporal dependencies due to its recurrent abilities, such as memory cells1. In this way, the LSTM can consider previous training examples on its forecasting process, creating a better understanding of the past data and a more robust combination process.

It is crucial to remark that both single and hybrid models struggled to forecast extreme points. This issue is a challenging task in the time series literature^12^. Another is the absence of sufficient extreme cases in the training set, which can bias the training process towards more regular cases and the applied target metric (MSE). The hybrid system’s computational effort is the sum of the costs of modeling its counterparts and depends on each model’s parameters set. The computational cost can be minimized in the test phase, parallelizing the time series and residual forecasting.

For future works, we intend to improve the accuracy of the hybrid systems to better forecast extreme values by automatically searching for the most suitable combination function. Besides, different base models, such as convolutional neural networks, echo state networks, and decision trees for regression, can be investigated.

## Supplementary Information

Supplementary Information.
