# Supplementary material for: Hybrid systems using residual modeling for sea surface temperature forecasting
Source: Sci Rep. 2022 Jan 11;12:487. doi: 10.1038/s41598-021-04238-z (PMC8752630; doi:10.1038/s41598-021-04238-z)
Supplement: Supplementary file 1 — Supplementary Information. [file 41598_2021_4238_MOESM1_ESM.pdf]

Supplementary Information

Hybrid Systems using Residual Modeling for Sea Surface Temperature Forecasting

Paulo S. G. de Mattos Neto, George D. C. Cavalcanti, Domingos S. de O. Santos Júnior and Eraylson. G. Silva*
***Centro de Informática, Universidade Federal de Pernambuco, Recife, Pernambuco, Brazil**

Supplementary Analysis

Tables S.1 and S.2 show the MSE values for the training, validation, and test sets obtained in each phase of the perturbative and NoLiC approaches, respectively. In the perturbative approach, the number of perturbations was defined as the last one that improved the value of MSE on the validation set compared to the previous perturbation. So, the perturbative method with the SVR employed three perturbations, while the LSTM model used two perturbations. Tables S.1 and S.2 show that, in general, there is a relationship between MSEs of the validation and test phases. In general, a correction that improved the MSE in the validation set compared to the antecedent perturbation also improved the MSE in the test set. This behavior shows that the ML model was adequately trained and, therefore, able to generalize unseen test patterns.

Table S.1. MSE values of the perturbative approach for the training, validation, and test sets attained to each perturbation.

Model	Dataset	Phase	P0	P1	P2	P3	P4
SVR	S1	Training	2.20E-04	1.58E-04	6.31E-03	4.62E-03	7.66E-04
		Validation	4.14E-03	8.44E-04	8.44E-04	8.44E-04	9.63E-04
		Test	3.78E-04	6.89E-04	6.89E-04	6.89E-04	6.89E-04
	S2	Training	2.11E-04	4.93E-04	1.15E-04	2.08E-04	1.65E-04
		Validation	2.24E-04	1.28E-04	1.27E-04	1.26E-04	1.72E-04
		Test	1.01E-02	1.08E-04	1.08E-04	1.08E-04	1.58E-04
	S3	Training	2.60E-04	2.51E-04	2.71E-04	2.66E-04	8.61E-04
		Validation	2.02E-03	7.98E-04	7.97E-04	7.95E-04	7.96E-04
		Test	2.58E-03	1.05E-03	1.05E-03	9.38E-04	9.26E-04
LSTM	S1	Training	5.40E-04	3.51E-02	1.54E-03	1.47E-03	-
		Validation	3.69E-02	1.35E-02	1.29E-03	1.45E-03	-
		Test	5.06E-03	6.78E-04	6.89E-04	6.88E-04	-
	S2	Training	8.90E-05	1.80E-04	1.89E-04	1.86E-03	-
		Validation	2.88E-04	2.36E-04	2.35E-04	2.39E-04	-
		Test	8.30E-03	1.58E-04	1.08E-04	1.59E-04	-
	S3	Training	5.20E-04	1.13E-03	1.04E-03	1.15E-03	-
		Validation	9.05E-04	8.90E-04	4.27E-04	6.13E-04	-
		Test	1.15E-03	7.85E-04	7.74E-04	7.91E-04	-

Table S.2. Results in terms of MSE for the training, validation, and test sets attained in each step of the NoLiC method.

Model	Dataset	Phase	P0	P1	Combination
SVR	S1	Training	2.20E-04	1.58E-04	8.80E-05
		Validation	4.14E-03	8.44E-04	1.42E-04
		Test	3.78E-04	6.89E-04	6.71E-04
	S2	Training	2.11E-04	4.93E-04	9.20E-04
		Validation	2.24E-04	1.28E-04	2.78E-04
		Test	1.01E-02	1.08E-04	1.01E-03
	S3	Training	2.60E-04	2.51E-04	3.26E-04
		Validation	2.02E-03	7.98E-04	5.98E-04
		Test	5.06E-03	6.78E-04	3.97E-04
LSTM	S1	Training	5.40E-04	3.51E-02	8.50E-04
		Validation	3.69E-02	1.35E-02	8.81E-04
		Test	8.30E-03	1.58E-04	1.31E-04
	S2	Training	8.90E-05	1.80E-04	1.62E-04
		Validation	2.88E-04	2.36E-04	1.84E-04
		Test	8.30E-03	1.58E-04	8.82E-03
	S3	Training	5.20E-04	1.13E-03	8.67E-04
		Validation	9.05E-04	8.90E-04	7.30E-04
		Test	1.15E-03	7.85E-04	7.74E-04

Figure S.1 shows the learning curve of the perturbative and NoLiC approaches employing the LSTM model for S1, S2 and S3 data sets. In these figures, the MSE values obtained by LSTM model in the training and validation sets can be compared. It is possible to note that for both approaches the LSTM model converged to a close MSE values in the training and validation sets. This behavior shows that the model was suitably trained, being able to generalize for new unseen test patterns.

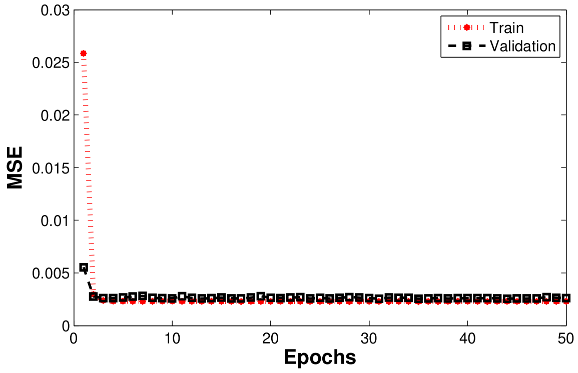

(a) Convergence curve of the perturbative approach using LSTM for S1 series.

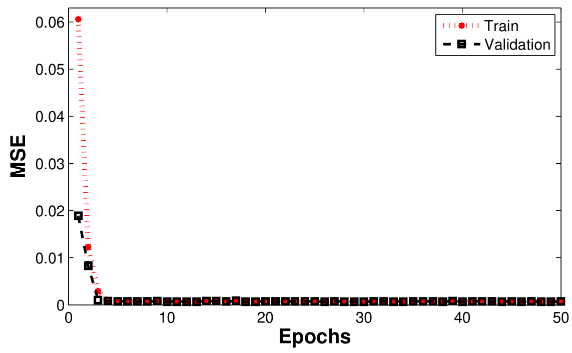

(b) Convergence curve of the NoLiC method using LSTM for S1 series.

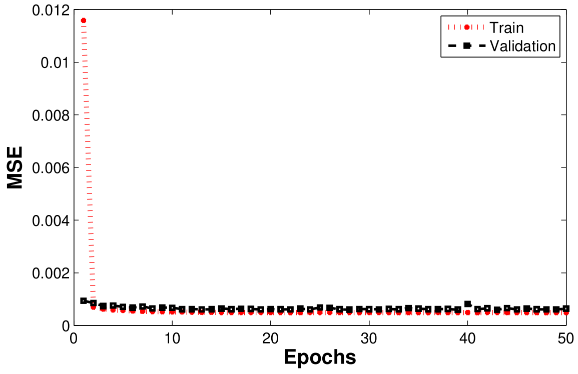

(c) Convergence curve of the perturbative approach employing LSTM for S2 series.

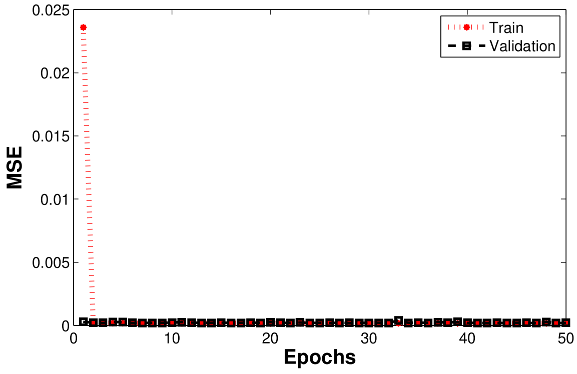

(d) Convergence curve of the NoLiC method employing LSTM for S2 series.

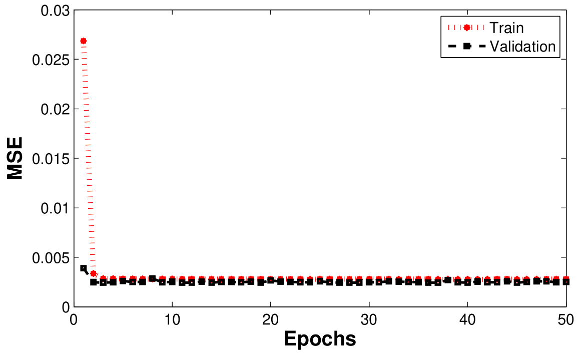

(e) Convergence curve of the perturbative approach with LSTM for S3 series.

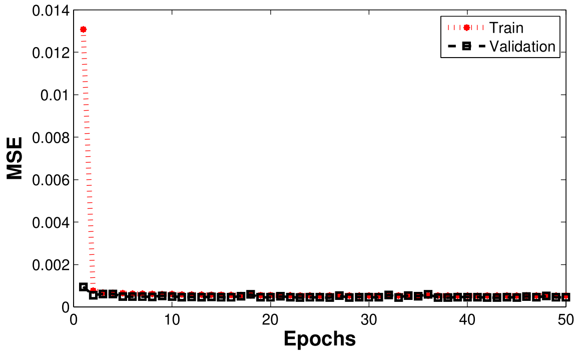

(f) Convergence curve of the NoLiC method employing LSTM for S3 series.

Figure S.1. Convergence curve of the perturbative and NoLiC approaches using the LSTM model for analyzed data sets (S1 (a - b), S2 (c - d) and S3 (e - f)).
